# 649. A Quality Improvement Project on Real-time Tracking of Acute Burn Wound Closure

**DOI:** 10.1093/jbcr/irag033.486

**Published:** 2026-04-06

**Authors:** David Crawford, Irene C Biallias, Julia Stehfest, Robert Beerman, Jolanda Tate, Paul Comish, Carolyn B Blayney, Tam N Pham

**Affiliations:** Regional Burn Center at Harborview Medical Center, Seattle, WA; University of Washington Medicine, Lynnwood, WA; University of Washington Harborview Medical Center, Seattle, WA; University of Washington Harborview Medical Center, Seattle, WA; University of Washington Harborview Medical Center, Seattle, WA; University of Washington, Seattle, WA; Regional Burn Center at Harborview Medical Center, Seattle, WA; University of Washington Harborview Medical Center, Seattle, WA

## Abstract

**Introduction:**

The American Burn Association (ABA) recently added outpatient outcomes to the Burn Care Quality Platform (BCQP). In response, our center implemented a quality improvement (QI) project to integrate inpatient and outpatient data to enhance our registry. Here, we report on the progress to-date with real-time capture of wound closure via Electronic Health Record (EHR) integration.

**Methods:**

A working group was established consisting of inpatient and outpatient clinical leadership, senior medical direction, and subject-matter experts in database architecture/data engineering, and software engineering. A crosswalk analysis was performed to harmonize the EHR with the new BCQP data dictionary. To adjudicate and standardize EHR and BCQP data points, projects were launched to integrate inpatient and outpatient data field completion. A specific integration was created between weekly burn diagram completion by bedside staff, outpatient staff, and EHR reporting. Wound-level data from 5 recent patients was abstracted as an exemplar of the impact of the enhanced data collection.

**Results:**

All 16 BCQP outpatient outcome measures were identified as part of standard data collection, but none were immediately accessible via EHR reporting. 11 were found in paper/clinic notes, and the remaining 5 were stored within the EHR back-end but their location varied. Refinements to inpatient data handling were successfully implemented to a level above BCQP requirements. The real-time reporting on wound status exemplar data (figure) demonstrate that both initial burn size and closure status are not well correlated with discharge.

**Conclusions:**

This project demonstrates the feasibility of integrating inpatient, outpatient, and long-term outcome data within EHR. Ongoing implementation requires tight collaboration between multiple hospital teams. An extension of this project to enhance EHR reporting capacity beyond BCQP requirements in all dimensions is currently underway.

**Applicability of Research to Practice:**

Inclusion of real-time outcome tracking within the clinical workflow.

**Funding for the study:**

N/A.

